# Supplementary material for: Evaluation of an online intervention for improving stroke survivors’ health-related quality of life: A randomised controlled trial
Source: PLoS Med. 2022 Apr 19;19(4):e1003966. doi: 10.1371/journal.pmed.1003966 (PMC9017949; doi:10.1371/journal.pmed.1003966)
Supplement: S1 Table — (DOCX) [file pmed.1003966.s002.docx]

| *Variable* | *Statistic/Category* | *Complete (n=356)* | *Drop out (n=43)* | *P-value* |
| --- | --- | --- | --- | --- |
| Age | n | 355 | 43 |  |
|  | mean (SD) | 68 (12) | 65 (13) |  |
|  | median (min, max) | 70 (20, 91) | 66 (41, 93) | 0.1069 |
| Sex | Female | 125 (35%) | 14 (33%) | 0.8658 |
|  | Male | 231 (65%) | 29 (67%) |  |
|  | Missing | 0 | 0 |  |
| State | NSW | 37 (10%) | 4 (9.3%) | 0.8734 |
|  | QLD | 155 (44%) | 22 (51%) |  |
|  | SA | 1 (0.3%) |  |  |
|  | TAS | 20 (5.6%) | 1 (2.3%) |  |
|  | VIC | 140 (39%) | 16 (37%) |  |
|  | WA | 3 (0.8%) |  |  |
|  | Missing | 0 | 0 |  |
| Stroke type | Don''t know (Don''t read out) | 13 (3.7%) | 5 (12%) | 0.0773 |
|  | Stroke | 216 (61%) | 23 (53%) |  |
|  | Transient Ischaemic Attack (TIA) | 127 (36%) | 15 (35%) |  |
|  | Missing | 0 | 0 |  |
| Country of birth | Australia | 273 (77%) | 34 (79%) | 0.0852 |
|  | England | 33 (9.3%) | 2 (4.7%) |  |
|  | New Zealand | 13 (3.7%) | 5 (12%) |  |
|  | Other | 36 (10%) | 2 (4.7%) |  |
|  | Missing | 1 | 0 |  |
| Indigenous status | Aboriginal and/or Torres Strait Islander | 1 (0.3%) | 2 (4.7%) | 0.0324 |
|  | Neither | 351 ( 99.7%) | 41 (95%) |  |
|  | Missing | 4 | 0 |  |
| Walk on admission | Yes | 197 (55%) | 23 (53%) | 0.9342 |
|  | No | 136 (38%) | 17 (40%) |  |
|  | Unknown | 23 (6.5%) | 3 (7.0%) |  |
|  | Missing | 0 | 0 |  |
