# Supplementary material for: Evaluation of an online intervention for improving stroke survivors’ health-related quality of life: A randomised controlled trial
Source: PLoS Med. 2022 Apr 19;19(4):e1003966. doi: 10.1371/journal.pmed.1003966 (PMC9017949; doi:10.1371/journal.pmed.1003966)
Supplement: Attachment — Submitted filename: PLOSMed Response Letter_240122.docx [file pmed.1003966.s003.docx]

Tuesday 14 February 2022

Callam Davidson

Associate Editor

PLOS Medicine

Dear Mr Davidson,

Thank you for your email dated 10^th^ January 2022 and the opportunity to revise and resubmit our manuscript PMEDICINE-D-21-04741R1 *“An online intervention for improving stroke survivors’ health related quality of life: a randomised controlled trial”*. We thank the reviewers for their thoughtful comments and believe that the manuscript is strengthened as a result of changes made. Please find below our detailed responses to reviewer suggestions.

**REQUESTS FROM THE EDITORS:**

**Abstract**

1. **Please report your abstract according to CONSORT for abstracts, following the PLOS Medicine abstract structure.**

The abstract has been updated according to the CONSORT for abstracts statement.

**Abstract Methods and Findings:**

2. **Please include the population and setting and provide a summary of the population demographics.**

The abstract has been updated to include and/or clarify and highlight these details.

3. **Please specify who was blinded to the intervention and control.**

The abstract has been updated to include and/or clarify and highlight these details.

4. **Please state that analysis was intention to treat (in both the abstract and the methods).**

The abstract (and methods) has been updated to include and/or clarify and highlight these details.

5. **Please provide the number of participants and the number of participants lost to follow up in each group.**

The abstract has been updated to include and/or clarify and highlight these details.

6. **Please include relevant p values when quantifying your results.**

The abstract has been updated to include and/or clarify and highlight these details.

7. **Please include the important dependent variables that are adjusted for in the analyses.**

The abstract has been updated to include and/or clarify and highlight these details.

8. **In the last sentence of the Abstract Methods and Findings section, please describe the main limitation(s) of the study's methodology.**

The abstract has been updated to include and/or clarify and highlight these details.

**Abstract Conclusions**

9. **Please interpret the study based on the results presented in the abstract, emphasizing what is new without overstating your conclusions.**

The abstract has been updated to include and/or clarify and highlight these details.

**General**

10. **Please remove the table of abbreviations and instead define abbreviations on first use.**

This change has been made.

11. **At this stage, we ask that you include a short, non-technical Author Summary of your research to make findings accessible to a wide audience that includes both scientists and non-scientists. The Author Summary should immediately follow the Abstract in your revised manuscript. This text is subject to editorial change and should be distinct from the scientific abstract. Please see our author guidelines for more information:** [**https://journals.plos.org/plosmedicine/s/revising-your-manuscript#loc-author-summary**](https://journals.plos.org/plosmedicine/s/revising-your-manuscript#loc-author-summary)

A non-technical Author Summary has been added following the Abstract in the manuscript.

12. **Citations should be in square brackets, and preceding punctuation.**

Citations have been updated to the appropriate format.

**Methods and Results**

13. **Thank you for completing the CONSORT checklist - please update to use section and paragraph numbers, rather than page numbers (as these are likely to change during the revision process).**

The CONSORT checklist has been updated to use section and paragraph numbers.

14. **Please define the abbreviations in Figure 1.**

Definitions have been added to the Figure 1 legend.

15. **Please define "lost to follow-up" as used in this study. Other reasons for exclusion should be defined.**

This has been defined in ‘Design’ paragraph of the Methods section.

16. **The protocol states that the research team ‘will monitor the ethics and safety of the trial’ – please confirm whether any relevant safety events were reported.**

No relevant safety events were reported over the course of the trial. This statement has been added to the first paragraph in the ‘Results’ section.

17. **Please provide the actual numbers of events for the outcomes, not just summary statistics or ORs.**

Actual numbers of events for the outcomes have been provided.

18. **Please define the abbreviations in Table 3.**

Abbreviations have been defined in Tables 2 and 3.

**Other**

19. **All content on page 16 (author contributions, funding, and disclosures) can be removed as this information will be captured as metadata based on your responses to the submission form.**

This change has been made.

20. **Please only use et al. after listing the first six authors in your references and use the journal name abbreviations found in the National Center for Biotechnology Information (NCBI) databases. See here for further information:** [**https://journals.plos.org/plosmedicine/s/submission-guidelines#loc-references**](https://journals.plos.org/plosmedicine/s/submission-guidelines#loc-references)

The references have been updated to the correct format.

**A NOTE TO THE EDITOR FROM THE AUTHORS**

In revising the manuscript it was noted that the variation in exclusion criteria was not included in the original manuscript submitted for review. This has been added to the final sentence of the ‘Recruitment’ paragraph in the Methods section and noted on the CONSORT checklist.

**REVIEWER 1: STATISTICAL REVIEWER**

**Major comments**

1. **Outcomes, page 8: I found it a bit confusing about what the primary outcome was - the EQ VAS is mentioned in the protocol paper although not clearly as the primary outcome; it's not mentioned in the ANZCTR (just EQ-5D-5L). I'm not an expert in the EQ-5D measurement which might explain my confusion, but I would welcome clarification on whether the specific primary outcome used in this paper was unambiguously pre-defined. This is especially true as EQ VAS results are significant but EQ-5D-5L results are not (although not far off)..**

The reviewer is correct in that the primary outcome in the protocol paper was health-related quality of life as measured by EQ-5D-5L. It does get confusing as the EQ-5D-5L consists of two pages/parts: 1) the EQ-5D descriptive system and, 2) the EQ visual analogue scale (EQ-VAS). However, these two parts do not form a single score. Thus, at the time of formalising the statistical analysis plan with the independent statisticians (which occurred before the end of data collection and prior to analysis of any data) it was advised and decided to proceed with one single primary outcome measure (EQ-VAS) rather than dual (EQ-VAS and EQ-5D) primary outcomes. The EQ-VAS was selected as the primary outcome measure as the sample size calculations had previously been made using EQ-VAS as the outcome guide.

A statement to this effect has been added to the ‘Outcomes’ paragraph in the Method section of the manuscript.

**Minor comments**

2. **Statistical analysis/Results: I note there was quite strong difference in missing data rates between arms. Since it's possible that the missing data may be missing not at random (it is plausible that individuals with worse outcomes were more likely to be lost to follow-up), are the authors concerned that this may explain the significant difference in results? This is especially true as the intervention did not significantly improve the behaviours that would be the mechanism to improving QoL. It may be useful to demonstrate that a large difference in missing outcomes (compared to computed outcomes assuming MAR) would be required to explain the significant results (a tipping point sensitivity analysis**

Thank you for the suggestion. We have conducted a tipping point analysis to assess the robustness of HRQoL to MAR. The results were robust to the assumption the data were MAR, however the results were not robust to an assumption that the difference in HRQoL between those with complete vs missing data was at least 3 points. We have updated the abstract, statistical analysis and discussion with these results.

3. **Abstract: It is not clear if the reported median and 95% CI is the improvement in the intervention group or the difference between arms. I note later that it is the difference - I would recommend making this clearer. Please add the p-value for significance.**

This has been clarified and the p-value added to the abstract.

4. **Abstract: Assuming the secondary outcomes listed are all of the secondary outcomes, I'd recommend changing 'included' to 'were'.**

This change has been made.

5. **Abstract: As this was a prospectively powered trial it is appropriate to provide p-values in addition to the confidence intervals.**

The p-values have now been added to the abstract.

6. **Statistical analysis, page 9: I understand the reason for using quantile regression instead of the analysis specified in the protocol, but I would recommend that it is highlighted as a difference to the protocol.**

The difference to the protocol has been highlighted as suggested in the third sentence of the second paragraph in the ‘Statistical analysis’ section of the Method.

7. **Page 11 - as per comment 3, please make clearer this is the between group difference and add the p-value.**

Clarification and the p-value has been added to the second sentence of the second paragraph in the Results section.

**REVIEWER 2**

1. **Incomplete information provided for funders (e.g., websites).**

This information has been added to the submission form and should now be captured as part of the metadata.

2. **In the abstract and introduction, it would help to define or make clear the difference between the primary and secondary HRQoL outcomes.**

In the abstract clarification has been made to indicate EQ-VAS is a self-rated health outcome and EQ-5D is a descriptive health state. The difference between the two rating components that form the EQ-5D-5L measure used to assess HRQoL have been elaborated on in the Methods section.

3. **Methods: How was “sufficient facility in Internet use” determined?**

Participants were required to either have or have access to an internet-enabled device (e.g. phone, computer, tablet device) or be willing to use public internet services (e.g. public library). This clarification has been added to the ‘Recruitment’ paragraph in the Methods section.

4. **The inclusion criteria state acute stroke or TIA while randomization was stratified by type of stroke defined as stroke TIA or other. Please clarify what is meant by “other.”**

The term ‘other’ has been amended to ‘don’t know’. This category was for participants who were unsure of the clinical classification of their stroke event. The data for ‘stroke type’ is presented in Table 1.

5. **Please clarify what additional “prompts” were used in the intervention group and frequency.**

This clarification has been added to the first paragraph of the ‘Methods: Intervention’ section. The intervention group received fortnightly text messages to remind them to use the online P2S program. These reminders were combined with the message to update contact details so that the intervention group were contacted fortnight 1: program use reminder; fortnight 2: program use + update contact details reminder.

6. **Was “time since stroke” considered during data analysis?**

Time since stroke was not considered during data analysis. The trial was designed to recruit stroke survivors in a specified time period post-stroke (i.e. 6 – 36months).

7. **Were data collected on intervention usability (e.g., number of times accessed the intervention and what resources or modules were accessed, perhaps some more than others)?**

Unfortunately, the software used to develop the program and the platform on which it was hosted were quite rudimentary and the backend data did not allow determination of comprehensive process measures. We have included the aggregate summary of modules accessed by those who did access the intervention in the 2^nd^ paragraph of the Results section.

8. **Discussion: Any differences noted between those that completed the study and those that were lost to follow-up? Perhaps higher rates of depression and anxiety in those lost to follow-up which was significant in the intervention arm. Same question of the 20% in the intervention arm that did not access or engage in the online program during the intervention time frame. Would also help to understand the reasons why these individuals did not access the program (e.g., technical difficulties etc.).**

A further comment has been added to paragraph 7 of the discussion section explaining that there were no demographic variable differences between participants missing and not missing at follow-up. We had already included the outcome variables in the imputation, this should account for any differences between dropouts in the analysis. The same applies to the imputation analysis accounting for differences between those accessing vs not the intervention. Reasons for not accessing the program were not collected. In development and pilot testing the participant feedback around challenges to using the program focused on capability to use computers and internet access. This was addressed in the trial by setting the eligibility criteria as stroke survivors who had access to computer/internet and were willing to use it for an online program.

**REVIEWER 3**

**Abstract**

1. **First sentence implies this is a study about secondary prevention-which is not consistent with the second sentence about quality of life.**

The first sentence has been removed to streamline the abstract and more strictly adhere to the CONSORT for Abstracts guidelines.

2. **Surveys are mentioned in the abstract-presumably these were the outcome questionnaires? If so can they clarify this please?**

The term ‘surveys’ has been changed to ‘outcome assessments’ to clarify.

3. **Primary outcome-should this be the 'change in VAS between baseline and 6 months' rather than the VAS?**

We have updated the abstract to read: ‘*The primary outcome was HRQoL as measured by the EQ-VAS (self-rated global health); the outcome was assessed for differences between treatment groups at follow-up, adjusting for baseline measures.*’

**Main paper**

4. **The rationale for an intervention preventing stroke recurrence having an effect on quality of life (as the primary outcome) needs to be made more clearly.**

HRQoL is a commonly used outcome measurement tool in stroke trials. The healthy lifestyles “P2S” intervention was developed to meet the health information needs and behaviour change challenges of stroke survivors. As we’ve outlined in the introduction many stroke survivors do not receive the necessary information on health risk behaviours and lifestyle management to improve recovery and contribute to reducing the risk associated with recurrent stroke. Improvement of these risk factors is often associated with improvement in health-related quality life. Over the past decade or more HRQoL has become an important outcome in stroke research as it is a multidimensional construct that encompasses many aspects such as physical, mental, and social health of the individual’s life. All of these small steps are in the direction of reducing risk of recurrence, however that overall outcome is not to scale for an intervention such as this. Assessing HRQoL, a measure that is related to all of the key modules/behaviours targeted by the online program and seen as critically important by survivors themselves, is a logical, practical and acceptable fit. We have made edits to the introduction section of the manuscript to highlight this.

5. **What was the rationale providing a list of internet links in the control group-was this an 'active control' or 'usual care'.**

The control group received minimal ethical care. As stated in the protocol the use of an active control condition was chosen as it balances exposure to online health programs and minimises the likelihood of changes in the treatment group due to non-specific therapeutic factors.

6. **Power calculations: 6 point difference in EQ-VAS: why was this chosen and is it of any clinical relevance?**

A 6-point difference in EQ-VAS is clinically significant in terms of improvement in quality of life, disability and clinical outcomes and was considered achievable based on similar secondary prevention pilot studies with stroke survivors.

7. **10.8% were lost to follow-up. The authors could describe how they attempted to minimise loss to follow-up, as this level of loss has the potential to introduce bias into the results.**

A description of the strategies used to reduce loss to follow-up has been added to the ‘Randomisation and data collection’ section of the Methods. The authors had initially planned for a 40% attrition rate based on the results of the pilot data specifically related to this style of intervention (referenced and cited in the manuscript).

8. **Physical activity was measured by self-report-to what extent was this valid? Did the authors consider directly measured activity, and if so, why was self-report chosen?**

The study was an online-only intervention and contact with participants was via telephone or email. Directly measured activity was beyond the scope of this study. All measures collected in the outcome assessments were self-report.

9. **Fatigue affects around 50% of stroke survivors and can have a major impact on quality of life. Why was fatigue not measured as an outcome? Is it plausible that an on-line programme like this could influence fatigue through a range of different mechanisms, and this might improve HRQUOL?**

Unfortunately, fatigue was not assessed as an outcome. A suggestion for future research has been added to the 5^th^ paragraph of the discussion.

Regards,

Dr Ashleigh Guillaumier

Heart Foundation Postdoctoral Research Fellow

T	+61 2 4033 5705

Ashleigh.Guillaumier@newcastle.edu.au
