# Supplementary material for: Evaluation of an online intervention for improving stroke survivors’ health-related quality of life: A randomised controlled trial
Source: PLoS Med. 2022 Apr 19;19(4):e1003966. doi: 10.1371/journal.pmed.1003966 (PMC9017949; doi:10.1371/journal.pmed.1003966)
Supplement: Attachment — Submitted filename: PLOSMed Response Letter_R2_160322.docx [file pmed.1003966.s004.docx]

Wednesday 16 March 2022

Callam Davidson

Associate Editor

PLOS Medicine

Dear Mr Davidson,

Thank you for your email dated 15^th^ March 2022 and the opportunity to revise and resubmit our manuscript PMEDICINE-D-21-04741R2 *“An online intervention for improving stroke survivors’ health related quality of life: a randomised controlled trial”*. We thank the editors and reviewers for their reviews of the manuscript. Please find below our detailed responses to editorial suggestions.

**REQUESTS FROM THE EDITORS:**

1. **Please update your title to “Evaluation of an online intervention for improving stroke survivors’ health related quality of life: a randomised controlled trial”.**

The manuscript title has been updated.

2. **Please cite your CONSORT checklist in the Methods, with a statement similar to ‘We reported our trial according to the Consolidated Standards of Reporting Trials (CONSORT) guidelines (S1 CONSORT Checklist)’.**

The ‘Design’ paragraph in the Methods section has been updated to include a sentence citing the CONSORT checklist.

3. **The term gender is used in Table 1 and Supplementary Table 1. Please consult http://www.who.int/gender/whatisgender/en/ and confirm that gender is correct term in this context (or whether sex would be more appropriate).**

The term ‘gender’ has been removed from Table 1 and Supplementary Table 1 and replaced with ‘sex’ to more accurately describe the variable measured.

4. **In the references, Journal name abbreviations should be those found in the National Center for Biotechnology Information (NCBI) databases.**

Journal names have been abbreviated.

5. **References 5, 11, and 40-42: Please update the DOI to remove https://doi.org/ (such that the format is consistent with e.g. reference 8).**

References 5, 11, and 40-42 have been updated as requested.

6. **Please add the date of citation to reference 49.**

The date of citation has been added to reference 49.

Regards,

Dr Ashleigh Guillaumier

Heart Foundation Postdoctoral Research Fellow

T	+61 2 4033 5705

Ashleigh.Guillaumier@newcastle.edu.au
